# Medical Videography Using a Mobile App: Retrospective Analysis

**DOI:** 10.2196/14919

**Published:** 2019-12-03

**Authors:** Julia C Cambron, Kirk D Wyatt, Christine M Lohse, Page Y Underwood, Thomas R Hellmich

**Affiliations:** 1 Mayo Clinic Alix School of Medicine Mayo Clinic Rochester, MN United States; 2 Division of Pediatric Hematology/Oncology Department of Pediatric and Adolescent Medicine Mayo Clinic Rochester, MN United States; 3 Division of Biomedical Statistics and Informatics Mayo Clinic Rochester, MN United States; 4 Legal Department Mayo Clinic Scottsdale, AZ United States; 5 Department of Emergency Medicine Mayo Clinic Rochester, MN United States

**Keywords:** photography, video recording, telemedicine, medical informatics applications

## Abstract

**Background:**

As mobile devices and apps grow in popularity, they are increasingly being used by health care providers to aid clinical care. At our institution, we developed and implemented a point-of-care clinical photography app that also permitted the capture of video recordings; however, the clinical findings it was used to capture and the outcomes that resulted following video recording were unclear.

**Objective:**

The study aimed to assess the use of a mobile clinical video recording app at our institution and its impact on clinical care.

**Methods:**

A single reviewer retrospectively reviewed video recordings captured between April 2016 and July 2017, associated metadata, and patient records.

**Results:**

We identified 362 video recordings that were eligible for inclusion. Most video recordings (54.1%; 190/351) were captured by attending physicians. Specialties recording a high number of video recordings included orthopedic surgery (33.7%; 122/362), neurology (21.3%; 77/362), and ophthalmology (15.2%; 55/362). Consent was clearly documented in the medical record in less than one-third (31.8%; 115/362) of the records. People other than the patient were incidentally captured in 29.6% (107/362) of video recordings. Although video recordings were infrequently referenced in notes corresponding to the clinical encounter (12.2%; 44/362), 7.7% (22/286) of patients were video recorded in subsequent clinical encounters, with 82% (18/22) of these corresponding to the same finding seen in the index video. Store-and-forward telemedicine was documented in clinical notes in only 2 cases (0.5%; 2/362). Videos appeared to be of acceptable quality for clinical purposes.

**Conclusions:**

Video recordings were captured in a variety of clinical settings. Documentation of consent was inconsistent, and other individuals were incidentally included in videos. Although clinical impact was not always clearly evident through retrospective review because of limited documentation, potential uses include documentation for future reference and store-and-forward telemedicine. Repeat video recordings of the same finding provide evidence of use to track the findings over time. Clinical video recordings have the potential to support clinical care; however, documentation of consent requires standardization.

## Introduction

### Background

**Figure 1:**
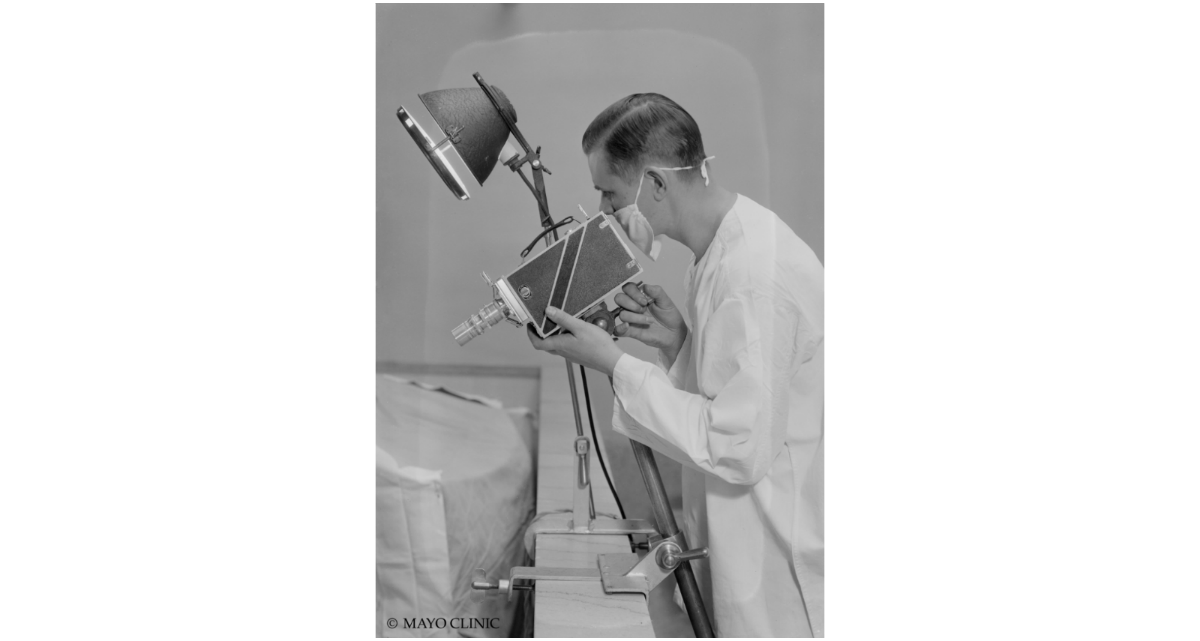
Videographer recording a surgical procedure at Mayo Clinic in Rochester, Minnesota, in March 1937. Copyright 1937 Mayo Foundation for Medical Education and Research. Used with the permission of Mayo Foundation for Medical Education and Research, all rights reserved.

Mayo Clinic’s institutional archives reference the use of video cameras to record surgeries as early as the 1930s using equipment that would be considered bulky by today’s standards. Historical photographs show videographers perched from balconies above operating theaters to record surgeries (Figure 1). As the process of video recording patients has become easier, the use of rich media for clinical documentation and diagnostic purposes has evolved.

At present, video cameras remain in use to capture medical procedures for the purposes of quality improvement, training, and research [1,2]. A recent systematic review on video recording open surgeries included 110 articles that discussed camera use to capture open surgery with miniature cameras, such as GoPro (GoPro, Inc.) and GoogleGlass (Alphabet, Inc) [3]. Outside of surgical specialties, video recordings are widely used to correlate clinical findings with electroencephalogram findings in patients with suspected seizures [4-6] and may also be used to study team dynamics and technical skills in emergency cardiopulmonary resuscitation [7]. In addition to asynchronous review of video recordings after they have been captured, live video can be used to conduct synchronous telemedicine encounters. For example, provider-to-provider telemedicine is used to guide neonatal resuscitation [8] and provider-to-consumer telemedicine is used for urgent care visits for minor illnesses [9].

At present, cameras available in smartphones arguably capture photographs and video recordings with better quality than most consumer-grade digital cameras that were available less than a decade ago. In a time when at least 77% of Americans own smartphones and more than 86% of physicians report using an electronic health record (EHR), merging both technologies presents an attractive and convenient opportunity to enhance point-of-care documentation [10,11]. Indeed, leading EHR vendors have seized this opportunity to integrate point-of-care image capture into their mobile apps, and institutions have developed home-grown apps for this purpose [12-14].

### Prior Work

We previously described the implementation of a mobile app (PhotoExam) for point-of-care clinical photography at Mayo Clinic [12], including its integration in the primary care setting [15]. Later evaluations revealed that the app’s primary use was not for teleconsultation but rather for improved documentation of physical examination findings within the EHR. Our initial assessment observed that residents and fellows represented the primary user base, with surgical specialties making up the largest number of users and dermatology accounting for most of the photographs [12]. Photographs were observed to be of acceptable quality, and the app’s release did not appear to be associated with a decrease in the use of traditional medical photography services. Our ongoing efforts are focused on assessing how point-of-care medical photography affects patient care–related outcomes.

The PhotoExam app has predominantly been used for its photography function, which allows authorized users to securely photograph a clinical finding and tag it with metadata (eg, anatomical site captured and description of finding). However, more recent versions of the PhotoExam app include capability to capture and upload video recordings.

### Goals

As it was unclear how the video recording function was being used in clinical practice and we were unable to identify literature about the use of point-of-care mobile clinical video recording technology elsewhere, we aimed to assess the following attributes of video recordings captured using the PhotoExam app: (1) who was capturing video recordings (in terms of specialty and work role), (2) what clinical findings were being captured on video recordings, and (3) was there a measurable impact of video recordings on patient care. Therefore, we retrospectively reviewed video recordings, their associated metadata, and patient records to assess the use and impact of the PhotoExam video recording feature.

## Methods

### The PhotoExam App

The PhotoExam app is available to health care providers at all Mayo Clinic sites via an internal *App Store* and is compatible with recent versions of iOS. Its release and updates were announced in internal communications (ie, staff newsletter). Any health care staff with access to the EHR can use the app while their device is securely connected behind the institutional firewall. Use of the app was not mandated by the institution. Although we are not aware of specific, formal departmental initiatives promoting use, we cannot rule out the possibility that departmental quality improvement initiatives may have encouraged use within specific specialties. Our anecdotal experience has been that clinical *champions* within a department often encouraged colleagues to use the app. The features and functionality of the video recording function are identical to those previously described for the photography function [12] except that only 1 video recording can be uploaded for each anatomic site (compared with multiple photographs per anatomic site), and the newest version automatically launches from within our EHR vendor’s proprietary mobile EHR app in a manner that hands off patient context, thereby obviating the need to manually search for the patient record within the PhotoExam app. Once the patient record is opened within the PhotoExam app, a hard stop verifies that the appropriate consent for photography or videography has been obtained according to departmental policies. The user is then permitted to capture a video recording using the device’s camera. The video recording is securely uploaded to a Digital Imaging and Communications in Medicine standard-compliant Digital Clinical Asset Management System. The captured video recordings are stored on the local device only temporarily until they are successfully uploaded or the user closes the app—whichever comes first—after which, they are permanently deleted from the user’s device in a manner that is compliant with the Health Insurance Portability and Accountability Act (HIPAA). Video recordings are not accessible by other apps on the mobile device. The app was not designed for patient use, and patients were not able to utilize the app to upload self-shot video recordings.

The users did not receive any formal training on practical aspects such as use case scenarios or how to capture clinically relevant video recordings with high fidelity. However, an internally accessible website provided technical support and instructions on the app’s use. We are unaware of formal integration of the app into medical training programs; however, users included residents and fellows in training.

### Human Subjects Protection

The study procedures were reviewed and approved by the Mayo Clinic’s institutional review board.

### Patient Selection and Data Collection

We queried the clinical asset management database to identify all video recordings taken using the PhotoExam app between April 15, 2016, (when the video recording feature first became available) and July 17, 2017. We excluded records corresponding to patients who refused the use of their medical records for research purposes, known test patients (ie, fictitious medical records within the production environment used for training and testing) and incidental video recordings (ie, user had not intended to record a video).

Employee work role and department were identified by cross-referencing a human resources database. These data were missing in the data source for a small number of users who were no longer employed by Mayo Clinic at the time of the query. Data available by review of the video recording and the patient medical records were extracted by 1 reviewer (JCC). Data were extracted into a data extraction form in REDCap [16] and exported for analysis.

### Quality Assessment

Lacking a standardized tool to assess the quality of provider-captured clinical video recordings, we adapted the quality assessment rubric used previously for assessing photographs taken using PhotoExam [12]. By consensus, we arrived at the following quality assessment items that were included in our rubric:

1. Does image quality or blurriness limit ability to see the area of focus?
2. Does the video recording objectively portray size using a ruler?
3. Does the video recording zoom in or out or move around to optimally characterize the finding as needed?
4. Is there sufficient lighting and color differentiation to see the area of focus?
5. Is the audio clear and audible (if present)?
6. Is the video recording image stable?
7. Is the video recording right side up?

A favorable score was awarded for an answer of *yes* on questions 2, 3, 4, 5, 6, and 7, and a favorable score was awarded for an answer of *no* on question 1. A quality score was calculated as a percentage of applicable favorable scores awarded on rubric items. For video recordings that included sound, the total score was calculated as a percentage of items out of 7. For video recordings that did not include sound, the total score was calculated as a percentage of items out of 6 because item 5 did not apply.

### Data Analysis

Continuous features were summarized with means and standard deviations when approximately normally distributed and with medians and interquartile ranges otherwise. Categorical features were summarized with frequency counts and percentages. For differences in quality score, referral generation, and consent documentation between specialties, analysis of variance and chi-square tests were performed across the most common user specialties (ie, orthopedic surgery, neurology, ophthalmology, and emergency medicine) with all other specialties grouped together as *other*. Throughout the paper, sample sizes for features with missing data or for subsets of interest are indicated in italics in parentheses. Results are reported at the video recording level—rather than patient level—unless otherwise specified. Statistical analyses were performed using SAS version 9.4 (SAS Institute). All tests were 2 sided, and *P* values less than .05 were considered statistically significant.

## Results

### Included Records

**Figure 2:**
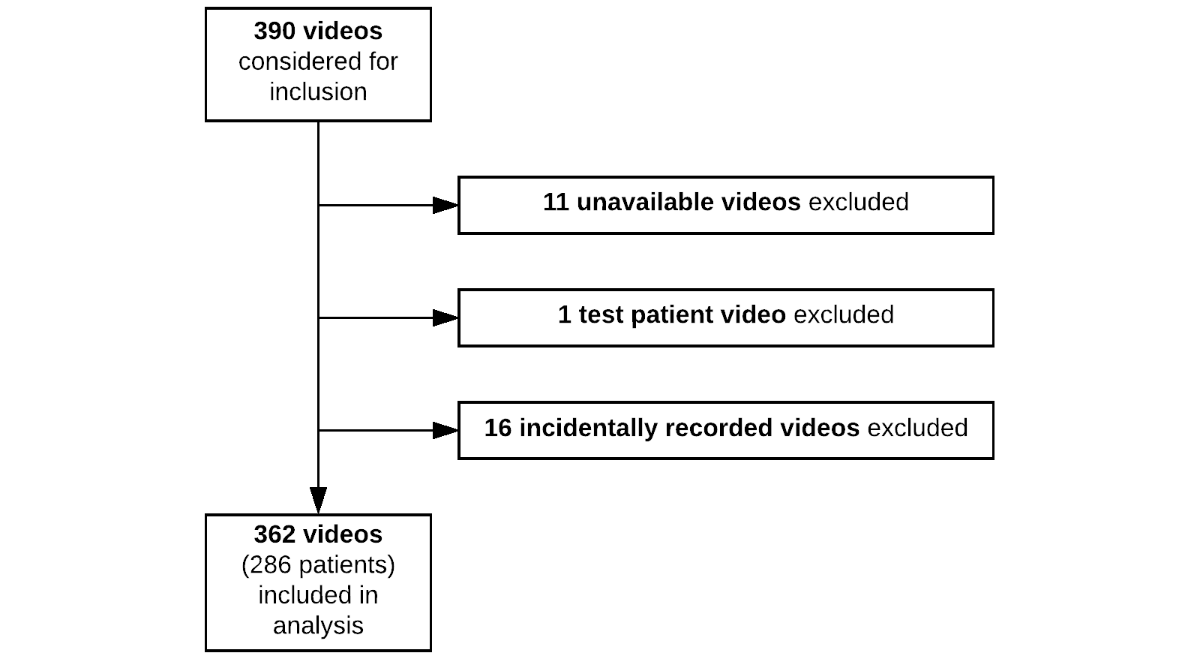
Patient record selection.

We identified 390 video recordings that were potentially eligible for inclusion. We observed that 11 video recordings were unavailable for review, 1 video recording corresponded to a *test patient* record that had not been identified in the initial screening process, and 16 video recordings appeared to be incidentally recorded. These video recordings were all excluded, yielding 362 video recordings of 286 patients that were included in the study (Figure 2).

### Demographics

**Figure 3:**
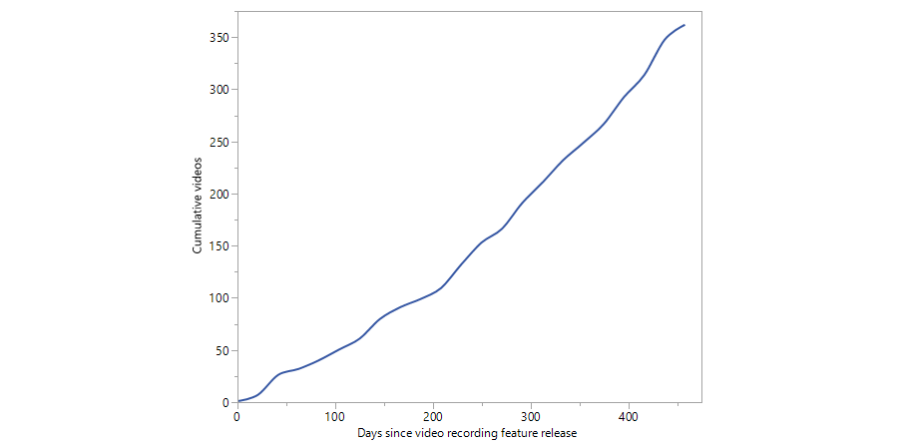
Videorecordings over time.

App use in terms of the number of clinical video recordings captured using the PhotoExam app over time is shown in Figure 3.

**Figure 4:**
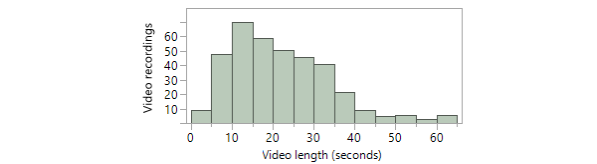
Distribution of videorecording lengths.

In terms of patient demographics, there was a slight predominance of males (58.0%, 210/362), and patients were largely white (88.6% [321/362]; Table 1). The most common site where video recordings took place was the Mayo Clinic Rochester campus, corresponding to nearly 4 out of 5 video recordings (79.0% [286/362]; Table 1). Most (70.7%, 256/362) video recordings took place in the outpatient clinic setting, followed by the inpatient hospital setting (20.4%, 74/362), emergency department (8.3%, 30/362), and, unexpectedly, patients’ homes (1.1% [4/362]; Table 1). The mean video length was 21 seconds (SD 12 seconds; Figure 4).

**Table 1:** Patient demographics and location of video recording, including work site and clinical setting.

Patient demographics (N=362)			Value
Age (years), mean (SD)			45.0 (24.6)
**Sex, n (%)**			
	Male		210 (58.0)
	Female		152 (41.9)
**Race, n (%)**			
	White		321 (88.6)
	Black/African American		10 (2.7)
	Asian		4 (0.2)
	Other		18 (5.0)
	Unknown		9 (2.5)
**Location of video recording**			
	**Work site, n (%)**		
		Mayo Clinic Rochester	286 (79.0)
		Mayo Clinic Arizona	43 (12.0)
		Mayo Clinic Florida	10 (2.8)
		Mayo Clinic Health System	23 (6.4)
	**Clinical setting, n (%)**		
		Hospital	74 (20.4)
		Clinic	256 (70.7)
		Emergency department	30 (8.3)
		Patient’s home	2 (0.5)

### User Demographics

The work role and primary clinical department of the provider who captured each video recording were assessed. The majority of video recordings were captured by attending physicians (54.1%; 190/351), followed by nurses (19.7%; 69/351) and residents/fellows (18.2%; 64/351). When assessed according to clinical department, approximately one-third (34.8%; 122/351) of video recordings were taken within orthopedic surgery, 21.9% (77/351) were taken within neurology, and 15.7% (55/351) of video recordings were taken within ophthalmology.

### Video Recordings and Photographs Within the Medical Record

**Figure 5:**
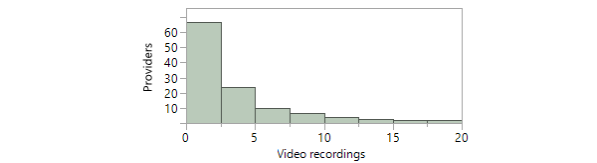
Distribution of provider experience level with videography. A single outlier who captured nearly 50 videorecordings is not included on the histogram.

**Figure 6:**
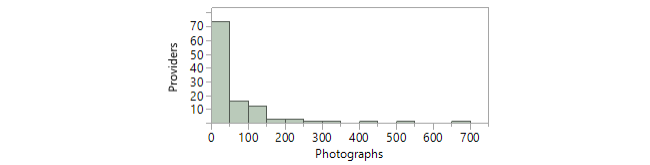
Distribution of provider experience with photography. A single outlier who captured over 1000 photographs is not included on the histogram.

To assess the extent of utilization of the app, we assessed the number of video recordings or photographs taken of the patient during each clinical encounter. To assess the provider’s experience level, we measured the cumulative number of video recordings and photographs the recording provider had previously captured using the app as of the time of each video recording. In most cases, only 1 video recording was recorded during the encounter and no photographs were captured (Table 2). Furthermore, providers had previously captured a median of 46.5 photographs and 3.5 video recordings using the PhotoExam app as of the time the video recording was recorded (Table 2). The distribution of provider experience level with videography and photography is graphically demonstrated in a histogram form in Figures 5 and 6, respectively**.**

**Table 2:** Photographs and video recordings associated with each clinical encounter and provider experience level at the time of use.

Associated photographs and video recordings		Value, median (IQR)
**Extent of app use during encounter**		
	Number of video recordings captured at visit	1 (1-2)
	Number of photographs captured at visit	0 (0-2)
**Provider experience level**		
	Cumulative number of video recordings captured by provider	3.5 (2-8)
	Cumulative number of photographs captured by provider	46.5 (14-111)

Patients included in 22 video recordings (7.7% of patients; N=286) were video recorded during subsequent clinical encounters, with 82% (18/22) of video recordings of these patients demonstrating the same finding seen in the index video.

Similarly, 19.2% (55/286) of patients’ video recordings were associated with additional photographs taken using the PhotoExam app. In 71% (39/55) of these cases, the photographed finding was the same as the video-recorded finding.

### Clinical Findings Recorded

Independent of the recording provider’s identified specialty, clinical findings were classified according to finding specialty categories. Table 3 summarizes the types of specific findings captured, stratified by finding category for the 4 most common specialty uses of orthopedic/musculoskeletal/physiatrics, neurologic, ophthalmologic, and dental, which accounted for more than three-quarters of all video recordings.

Table 4 includes additional details about the findings documented in the reviewed video recordings. Most of the video recordings (68.8%; 249/362) captured volitional movements by patients, and 29.6% (107/362) of them captured either passive movements initiated by the examiner or static findings. The vast majority (92.3%; 334/362) of video recordings captured physical examination findings; however, less common uses included demonstration of cares (eg, wound care), capture of externally captured media (eg, video recording originally captured on patient’s cellphone or portable ultrasound), and objects external to the patient (eg, foreign body). A variety of anatomic sites were captured, with the most common sites being the upper extremities (31.5%; 114/362) and the eyes (18.0%; 65/362).

**Table 3:** Specialty-specific findings included in the video recordings.

Specialty findings (N=362)		Value, n (%)
**Orthopedic/musculoskeletal/physiatrics**		130 (35.9)
	Range of motion	102 (28.2)
	Muscle strength test	4 (1.1)
	Other	38 (10.5)
**Neurologic**		80 (22.1)
	Motor function and balance	19 (5.2)
	Coordination test	17 (4.7)
	Cranial nerve test	7 (1.9)
	Mental status	5 (1.4)
	Reflexes	4 (1.1)
	Other	40 (11.0)
**Ophthalmologic**		58 (16.0)
	Extraocular movement	26 (7.2)
	Slit lamp examination	5 (1.4)
	Pupillary response	2 (0.6)
	Other	31 (8.6)
**Dental**		13 (3.6)
	Surgical prosthesis or device	11 (3.0)
	Other	2 (0.6)
Other findings		83 (22.9)

**Table 4:** General characteristics of the findings captured in video.

General finding characteristics (N=362)			Value, n (%)
**Volition**			
	Nonvolitional	107 (29.6)	
	Volitional	249 (68.8)	
	Both	6 (1.7)	
**Finding type**			
	Object outside patient	3 (0.8)	
	Capture of video recording on another device	5 (1.4)	
	Capture of imaging findings	11 (3.0)	
	Physical exam	334 (92.3)	
	Demonstration of care	9 (2.5)	
**Anatomic site captured**			
	Hand	75 (20.7)	
	Eye	65 (18.0)	
	Arm	39 (10.8)	
	Entire body/unspecified	44 (12.2)	
	Leg	29 (8.0)	
	Face	23 (6.4)	
	Mouth	15 (4.1)	
	Chest	14 (3.9)	
	Head	12 (3.3)	
	Other	46 (12.7)	

In 107 (29.6%; 107/362) video recordings, we noted that people other than the patient were incidentally captured. In 80 (74.8%; 80/107) of these, medical personnel were included, and in 31 (29.0%; 31/107) video recordings, other people who had accompanied the patient to the visit (ie, family or friends) were included. As both medical personnel and other people who had accompanied a patient to a visit could have been captured in the same video recording, these percentages add up to greater than 100%.

### Captured Audio

As the PhotoExam app allows the user to include or omit the recording of sound, we were interested in the inclusion and content of the recorded audio. Audio was only recorded in 118 (32.6%; 118/362) video recordings. In 36.4% (43/118) of cases, only background noise was captured, suggesting that audio did not need to be recorded in those cases. In 55.1% (65/118) of cases, the audio included provider instructions (such as commands to the patient to complete physical examination maneuvers), and in 11.0% (13/118) of cases, providers recorded commentary (eg, description) about the observed findings. In 28.0% (33/118) of cases, sounds made by the patient, which may be relevant to an observer, were captured.

Upon manual review of these 118 video recordings that included audio, the audio recorded findings were deemed to be the primary finding in 2.5% (3/118) of the video recordings, audio recorded and video recorded findings were considered equally important in 27.1% (32/118) of cases, and, in the remaining 70.3% (83/118), the video recorded finding was deemed the primary finding.

### Video Recording Quality

Our quality assessment revealed that most video recordings were acceptably in focus with adequate lighting, stability, and sound. However, video recordings frequently did not adequately demonstrate scale (ie, by using a ruler) or perspective (ie, by rotating the camera around clinical findings). The mean quality score across video recordings was 67.8 (SD 7.7)%. There was no significant difference in quality score when compared across specialties. Average quality scores ranged between 65.8% in emergency medicine and 68.5% in *other* specialties, with quality scores of ophthalmology, neurology, and orthopedic surgery falling within this range (*P*=.59).

### Consent

In 115 (31.8%; 115/362) video recordings, patient consent was clearly documented in the medical record: for 113 of these video recordings (98.2%), a signed media consent form was scanned into the medical record, and for 2 video recordings (1.7%), the provider documented in the clinical note that verbal consent was obtained. There was a significant difference between specialties in terms of the percentage of video recordings that were associated with explicitly documented consent within clinical notes or a signed media consent form (*P*<.001). Exploratory comparisons between individual specialties and the others revealed driving factors to be high rates in orthopedic surgery (53.3%, 65/122; *P*<.001 vs all others) compared with low rates in emergency medicine (13.3%, 4/30; *P*=.02 vs all others) and *other* (ie, not orthopedic surgery, neurology, ophthalmology, or emergency medicine) specialties (11.5%, 9/78; *P*<.001 vs all others).

### Clinical Impact

We first evaluated clinical notes to identify evidence of the clinical impact of video recordings. Disappointingly, only 44 (12.2%; 44/362) video recordings were referenced in the clinical notes corresponding to the visit, and in only 2 cases (0.6%; 2/362) the use of telemedicine with remote, asynchronous review of the video recording by another provider was explicitly documented in the clinical notes.

In 30 cases (8.3%; 30/362), an outcome of the visit was generation of a referral to a specialist. In the majority of these cases (20/30; 67%), the referral occurred on the same day. Of the 20 same-day referrals, 14 (70%) were specialist referrals made for patients seen in the emergency department. Overall, 16 total referrals were made in the emergency department, meaning that only 2 patients for whom a referral was generated in the emergency department were not seen by the specialist on the same day the referral was made. Neurology and plastic surgery accounted for the majority of the specialists consulted, contributing 10 and 6 referrals, respectively. There was a significant difference between departments in the proportion of video recordings associated with a referral (*P*<.001), with the main driver being a high rate of referrals made from the emergency department, where half of the video recordings were associated with a referral compared with 7% or fewer encounters for all other specialties.

### Qualitative Aspects and Real-World Use Cases

In this study, we categorized and summarized the myriad ways that health care providers have used video recordings to document clinical findings. One unfortunate consequence of aggregating data is that the qualitative richness of the video content becomes lost. To capture these uses, we therefore include a few qualitative observations to highlight innovative or interesting use cases. One coauthor (TRH) utilized the app to capture the work of the breathing of an infant with bronchiolitis who required hospital admission. Another coauthor (KDW) who was covering the pediatric ward was able to view the video from elsewhere in the hospital, and this was noted to facilitate the appropriate disposition of the patient to the intensive care unit.

Overall, 19 video recordings demonstrated *motor function and balance* in a variety of ways. Common use cases included capturing of abnormal gait (eg, ataxia), tremors, and dystonia. Face, letter, and number recognition exercises were recorded and used to document mental status. Ophthalmologic video recordings captured slit lamp examinations, which were generally conducted without the use of a magnifying objective.

Innovative uses included capturing radiographic videos (eg, fluoroscopy, echocardiography and other ultrasonography captured on portable machines and at outreach locations that utilize a different EHR), dressing of a wound and application of vacuum-assisted closure device (ie, intended to be instructive to other providers), recording brief procedures (eg, plantar corn removal) and capturing patient-provided video recordings (eg, capturing a video recording of a video playing on the patient’s phone in order to integrate it into the EHR).

## Discussion

To our knowledge, this is the first report in the literature to summarize the use of a point-of-care mobile app to capture and upload clinical video recordings to the EHR in a manner that is secure and HIPAA compliant.

### Principal Findings and Comparison With Prior Work

In our initial report on the use of the photography feature of the PhotoExam app, attending physicians captured more photographs than other user groups (29.5%; 6725/22,784), with nurses taking a similar number of photographs (28.3%; 6446/22,784). In this study, attending physicians and nurses were still the leading users, though there was a greater discrepancy between the percentage of video recordings captured by the top user groups when compared with photographs, with attending physicians and nurses capturing 54.1% (190/351) and 19.7% (69/351) of video recordings, respectively. With respect to specialty, our initial report on PhotoExam revealed that 54.1% (12,315/22,784) of photographs were captured within dermatology and 25.6% (5825/22,784) were captured within surgery. In contrast, leading user departments in this study were orthopedic surgery (33.7%; 122/362), neurology (21.3%; 77/362), and ophthalmology (15.2%; 55/362). This is not surprising as dermatology and surgery may be able to rely on static clinical findings, whereas orthopedic surgery, neurology, and ophthalmology commonly refer to active physical examination findings.

Analysis of usage patterns indicated steady growth in use over time, though the number of photographs taken using the app dwarf the number of video recordings. For example, in the first 8 months after the launch of the PhotoExam app, 22,784 photographs were captured compared with 148 video recordings in the 8 months following the addition of video recording functionality to the app [12]. There are several possible explanations for this discrepancy. One possibility is that users are not as aware of or familiar with the video recording feature. Another possibility is that many users may find the photography feature to be sufficient to capture clinical findings, thereby obviating the need to capture a video recording.

Most video recordings were recorded at the Mayo Clinic Rochester campus. This likely—at least partly—reflects differences in patient visit volumes between sites. The majority of video recordings were taken by attending physicians. However, nurses and residents/fellows also captured a significant number of video recordings. In comparison, in our previous study on the use of the app to capture photographs, attending physicians captured more photographs than other user groups (29.5%; 6725/22,784) but nurses took a similar number of photographs (28.3%; 6446/22,784) [12]. On the basis of review of the video recordings and medical records, as well as practical experience with the app’s use, nurses and residents/fellows may take video recordings on their own initiative or may be delegated by an attending physician to capture a video recording.

In addition to permitting moving images to be captured, video recordings also permit sound to be recorded. Common inclusions on audio tracks included provider commands (which allow the viewer to follow what is being asked of a patient) and a provider’s verbal description (to indicate what is being captured). Furthermore, sounds made by patients, which could be relevant to the clinical finding, were also captured.

In general, video recordings were of high quality, though many did not extensively demonstrate perspective by rotating about the clinical finding, and many did not explicitly demonstrate size/scale using an objective measuring device (ie, ruler). In retrospect, perspective may be less important for some findings (ie, seizure) than for others (ie, raised subcutaneous abscess) and therefore may be less relevant to consider when assessing these video recordings for quality. In addition, our rubric may have been excessively stringent by requiring the use of a ruler to be deemed as adequately demonstrating size/scale. Our previous use of this rubric for assessment of photography quality [12] did not strictly require size to be portrayed using a ruler—photographs could be considered to demonstrate size if other anatomical landmarks that infer size were included. In that study, 12.0% (12/100) of photographs did not adequately demonstrate size [12] compared with 99.4% (360/362) of video recordings in this study. In retrospect, many video recordings that we reviewed for this study were able to reasonably demonstrate size/scale by including an object of reference within the frame or by starting the video recording showing the patient’s entire body or recognizable landmarks and then slowly panning and zooming to the area of interest to capture size. Therefore, the use of overly stringent criteria to judge demonstration of size represents a limitation of the quality adjudication criteria used in this study. Furthermore, assessment of quality by only 1 reviewer was another limitation. In future studies, we will adjust the quality rating scale based on our observations, and we will consider reviewing at least a subset of video recordings in duplicate to ensure consistency in the quality assessment process.

Patient consent was not always clearly documented in the EHR. However, we are confident that patient consent was obtained for all video recordings because the app includes a hard stop (toggle switch and popup dialog box) that requires the user to attest that consent was obtained from the patient before being permitted to proceed with photography. One reason verbal consent may not have been clearly documented in clinical notes could be that providers may have thought that attesting that consent was obtained within the app was sufficient for documentation purposes. One possible improvement to the app would be to have the user select the method of consent (ie, verbal or written) and indicate the person providing consent (ie, patient, parent, guardian, and legal representative), which would then be documented with the video recordings. During the early stages of the app’s development, the idea of having patients sign the screen of the device to provide written consent was considered. However, this option was not pursued because providers’ personal mobile devices were often used and would ideally need be sanitized before having patients handle them. Furthermore, logistical issues relating to internal form processing and practical considerations of handing a patient an unlocked mobile device that contains protected health information and other private, personal information limited our ability to pursue this mechanism for consent documentation.

In many cases, people other than the patient were incidentally captured in video recordings. Most of the individuals incidentally captured on the video recordings appeared to be staff members, but others appeared to be family or friends who accompanied the patient. It is unclear whether these individuals also separately provided consent to be included in the video recordings or whether their consent was implied by facilitating video recording (ie, parent holding a child while the provider captures the video recording). From a legal standpoint, patients have a fundamental right to privacy that includes the right to consent to be photographed or video recorded. Staff, family, and friends do not have the same privacy rights. Courts have held that when individuals are in public areas, such as common areas within a hospital, they do not have reasonable expectations of privacy and can be photographed and, in most states, video recorded without the need for their prior consent. In a minority of states, a recording of a conversation requires the consent of every person in the conversation. If the video recording occurs in one of these *2-party* consent states, consent is required of each staff member, family member, or friend who is a party to the recorded conversation. However, consent can be expressed or implied based on the circumstances. As in the case of the parent holding a child, if the individual is aware of the video recording and continues to participate, consent may be implied.

We observed evidence that video recordings favorably affected clinical care. For example, we observed that video recorded findings were either video recorded or photographed at subsequent visits, suggesting that clinical findings were being tracked over time using the app and presumably referenced at those visits for comparison. In 8% of cases, a referral to a specialist was generated, and in two-thirds of these cases, referrals occurred the same day. Furthermore, video recordings captured in the emergency department were more likely to be associated with a referral than those captured within other specialties. Although it is tempting to speculate that the existence of a video recording within the EHR facilitated or expedited referrals, we do not have additional evidence to support this hypothesis. The authors’ anecdotal experience has been that consulting specialists find video recordings helpful because they allow specialists to gather important information and provide tentative management advice before seeing the patient in-person. Review of video recordings may also help specialists prioritize consultations or expedite care when appropriate (eg, notify the operating room staff of an anticipated procedure).

We were disappointed that providers only infrequently mentioned video recordings within clinical notes. In fact, when video recordings were mentioned in clinical notes, they were only mentioned in notes corresponding to the clinical encounter where the video recording was recorded and were not mentioned in subsequent follow-up visits. We had anticipated that other providers who see the patient in follow-up might reference previously taken video recordings. As noted above, we speculate that providers who captured repeat video recordings or photographs at follow-up visits viewed those video recordings, but we do not have evidence of this. It is unclear whether the absence of further references in follow-up and referred consultation notes reflects an incomplete search of the patients’ medical records, a lack of viewership by subsequent physicians, a simple omission on the part of the provider in documentation, or a lack of impact of the video recording on future medical care. Video recording access log audits would help demonstrate how often other providers view the video recordings.

### Strengths and Limitations

Strengths include that we captured data from a variety of sources, including photograph metadata, human resources records, manual review of video recordings, and review of clinical documentation. We also reviewed longitudinal data to identify when additional photographs or video recordings of the captured findings were obtained.

The major limitation was the use of a single reviewer to abstract data and review video recordings. Although we included patients seen at multiple sites within various settings, another limitation that limits the generalizability of our findings is that our patient population is not representative. Furthermore, we were unable to clearly associate video recordings with objective clinical outcomes. We systematically assessed the technical quality of video recordings; however, in this descriptive study, we did not include an assessment of suitability for clinical use. Conducting such an assessment in the course of our review presents several challenges. For one, the reviewer may not be familiar with the clinical requirements of a given subspecialty and may therefore not be appropriate to assess the suitability for clinical purposes. Second, the clinical utility may not be apparent or evident to those who are not directly involved in the patient’s care during a retrospective review (ie, third-party reviewer). In part, to address this aspect of the app’s use and assess the clinical impact of the app, we separately conducted Web-based user surveys (unpublished) that included questions about use cases and outcomes of photography. To keep the survey brief and maximize the response rate, the questions asked about the app in general and did not ask the same questions separately about the photography and videography features. Most users utilized the app exclusively to capture photographs—rather than video recordings—and, therefore, responses in general were in reference to photography. Therefore, the clinical utility of the video recording feature remains an area needing study.

### Areas of Future Research

Through the authors’ use of the app in clinical practice, a number of legal questions have arisen. Although it is out of the scope of this study to review them all, several of these questions are worth raising. For example, in video recordings where people other than the patient are included, have consent laws been violated? In some cases, consent for video recording may be implied—for example, if a parent holds a child in their lap and poses them to the video camera for photography, they are providing implied consent. However, if another patient is captured on a video recording while walking by, they have not provided implied consent. Even in cases where others may be incidentally included in video recordings, the HIPAA privacy rule permits certain incidental disclosures as long as reasonable safeguards are put in place to limit these disclosures. For example, policies, procedures, and training may be reasonable safeguards to limit incidental disclosures. In cases where others are incidentally included in recordings, other privacy rights beyond HIPAA may limit reuse of the video recording without the express consent of all parties included in the video recording.

If a recording is to be used in court proceedings, state laws may dictate consent requirements. For example, some states have *two-party consent* laws that require consent of parties involved to be used as evidence in court. Another legal consideration if the video recording is to be used for legal proceedings is maintenance of the chain of custody. The use of electronic log files that document the creation and any modifications to the video recording can help maintain the chain of custody.

### Conclusions

In conclusion, the video recording feature of the PhotoExam app has proven to be a versatile tool that has uses within many different specialties at Mayo Clinic. Clinical video recordings offer the potential to augment clinical documentation, support assessments at follow-up visits, and facilitate telemedicine. In particular, the ability to closely track patient examination findings over time offers the potential to more accurately document chronic and progressive conditions. This technology may be used to capture objective assessments of the efficacy of treatments and medical interventions over time, and it can facilitate collaboration among multiple members of a multidisciplinary patient care team.

## Abbreviations

EHR: electronic health record
HIPAA: Health Insurance Portability and Accountability Act
